# Elite female athletes’ experiences of symptoms of pelvic floor dysfunction: A systematic review

**DOI:** 10.1007/s00192-022-05302-6

**Published:** 2022-08-30

**Authors:** Elizabeth Culleton-Quinn, Kari Bø, Neil Fleming, David Mockler, Cinny Cusack, Déirdre Daly

**Affiliations:** 1 School of Medicine, Trinity College, Dublin, Ireland; 2 Discipline of Physiotherapy and Discipline of Occupational Therapy, Trinity College, Dublin, Trinity Centre for Health Sciences, James’s St., Dublin, D08W9RT Ireland; 3 Department of Sports Medicine, Norwegian School of Sports Sciences, Oslo, Norway; 4 Department of Obstetrics and Gynaecology, Akershus University Hospital, Lørenskog, Norway; 5 Medical Library, Trinity College, Dublin, Ireland; 6 Physiotherapy Department, Rotunda Hospital, Dublin, Ireland; 7 School of Nursing and Midwifery, Trinity College, Dublin, Ireland

**Keywords:** Elite athletes/sportswomen, Experiences, Pelvic floor dysfunction

## Abstract

**Introduction and aims:**

Pelvic floor dysfunction (PFD) is a collection of signs, symptoms and conditions affecting the pelvic floor and urinary incontinence (UI) is the most common type of PFD. Recent systematic reviews have indicated a higher prevalence of UI among female athletes compared to their non-athletic counterparts. To date, no review has been undertaken to investigate female athletes’ *experiences* of PFD. This review aims to offer insight and understanding, through aggregation, summary, synthesis and interpretation of findings from studies that report elite female athletes’ experiences of symptoms of PFD.

**Methods:**

The review protocol was registered in PROSPERO in August 2020. A systematic search was conducted in Embase, MEDLINE (OVID), Cochrane Library, CINAHL, PsycINFO and Web of Science for studies published in the English language reporting elite female athletes’ experiences of symptoms of PFD. This review included primary research studies that involved elite female athletes of any age or ethnicity.

**Results:**

Of the 1922 citations retrieved in the search, 32 studies met the methodological criteria for data extraction and analysis. Five main themes emerged: (1) triggers for symptoms of PFD; (2) strategies adopted by athletes to manage/mitigate symptoms of PFD; (3) impact on QOL/daily life; (4) impact on performance; (5) impact on emotions.

**Conclusions:**

The findings of this review suggest a need to further explore the experiences of PFD among elite female athletes and it is suggested that future research should adopt qualitative methods or incorporate a qualitative component.

## Introduction

Pelvic floor dysfunction (PFD) is a collection of signs, symptoms and conditions that affect the pelvic floor [1]. Urinary incontinence (UI), the PFD most commonly experienced by women, is defined as a ‘complaint of involuntary loss of urine’ and is a common complaint in women of all ages. The most common types of UI include stress urinary incontinence (SUI) and urgency urinary incontinence (UUI). The International Urogynecological Association (IUGA) and the International Continence Society (ICS) define SUI as the ‘complaint of involuntary loss of urine on effort or physical exertion (e.g., sporting activities), or on sneezing or coughing’ and UUI as ‘complaint of involuntary loss of urine associated with urgency’ [1]. Other symptoms of PFD include anorectal dysfunction (ARD), sexual dysfunction (SD), pelvic organ prolapse (POP) and pelvic pain [1, 2]. Many women find it embarrassing to discuss symptoms of PFD including continence problems with others and incontinence has been shown to negatively affect quality of life [3–5].

Mendes et al. [6] conducted a systematic review of qualitative evidence regarding adult women’s experiences of UI. Findings from the 28 included studies were grouped into eight themes in the areas of: cultural and religious backgrounds; effect on daily activities/social roles; knowledge and nature of symptoms; experiences of UI and sense of shame; negative effects on intimacy, sexuality and sexual function; UI seen as consequence of pregnancy/childbirth, aging or religious punishment; strategies adopted by women affected by UI; meeting of care needs and women’s personal preferences. The authors concluded that the preferences and expectations of women with UI should be considered and that the provision of healthcare should be personal and tailored. A need for additional research to improve the understanding of the impact of UI on the quality of life (QOL) of younger women was identified [6].

UI during exercise is not uncommon and a higher prevalence has been observed among athletes engaged in high-impact sports including running and jumping [7]. Rodríguez-López et al. [8] investigated the prevalence of UI in both female and male elite athletes and found an overall prevalence of 33% (45.1% in females, 14.7% in males) and that, whilst the prevalence of UI was 5.45 times greater in females, elite male athletes were also found to experience UI.

There have been a number of recent systematic reviews concerning PFD in female athletes [9–14]. However, the main aim of these reviews has been the investigation of the prevalence of UI in female athletes. Almousa and Bandin Van Loon [9] included a secondary aim of exploring the knowledge and attitudes of female athletes regarding UI [9] and de Mattos Lourenco et al. [10] discussed strategies adopted by the athletes to manage their UI. The reviews differ somewhat in their inclusion criteria regarding age and parity, but they all concluded consistently that there was a higher prevalence of UI among female athletes compared to non-athletes. Engaging in high-impact sports [9, 10, 12, 14] with longer hours of training [9] appears to be commonly cited as risk factor for UI, but to our knowledge, no review has been undertaken to investigate female athletes *experiences* of PFD. Studies have found that experiencing UI during elite sports may be a predictor of UI in later life [15] and also that elite athletes have been identified as an understudied population in the research into PFD and, in particular, ARD and POP and physical activity [16].

Whilst a scoping review of the research literature revealed limited qualitative research into the area of PFD among female athletes, such research may, potentially, yield further information regarding the impact of the symptoms of PFD on the female athletes’ sporting activities and their daily lives.

Therefore, this review aimed to offer insight and understanding, through aggregation, summary, synthesis and interpretation of findings from studies that report the experiences of symptoms of PFD in elite female athletes.

## Materials and methods

### Study design and protocol registration

This systematic review complied with the ‘Adapted PRISMA guidelines for reporting systematic reviews of qualitative and quantitative evidence’ [17, 18]. The review protocol was registered in PROSPERO in August 2020 and is available at https://www.crd.york.ac.uk/prospero/display_record.php?ID=CRD42020197330 [19].

### Search strategy and selection criteria

With the assistance of a medical librarian (DM), the electronic databases of Embase, MEDLINE (OVID), Cochrane Library, CINAHL, PsycINFO and Web of Science were searched, initially in May 2020 and subsequently in an updated search in January 2022 for studies that reported female athletes’ experiences of incontinence/symptoms of pelvic floor dysfunction. The search terms include the following: Wom?n, Femal*, urinar* continen* or incontinen*, Pelvic Organ Prolapse, Urinary leakage, leaking urine, vaginal wind, anal incontinen*, f?ecal incontinen*, Bladder leakage, bowel leakage, Flat* incontinen*, Physical* ADJ (activ* or inactiv* or exercise), recreational* ADJ (activ* or inactiv* or exercise), Exercis* ADJ (strenuous OR vigorous OR moderate)), Activit* ADJ (strenuous OR Vigorous OR moderate OR Leisure), sport*, Participat*.ti, ab., Modif*.ti, ab., Stop*.ti, ab., Adapt*.ti, ab., Change*.ti, ab., Limit*.ti, ab., Abandon .ti, ab., Ceas*.ti, ab., Barrier.ti, ab., Impact.ti, ab., Affect.ti, ab.

This review included primary research studies published in the English language that reported elite female athletes’ experiences of symptoms of PFD. Inclusion criteria were: (1) studies that involved female athletes of any age or ethnicity and studies that included both female and male athletes together when data on female athletes could be extracted; (2) studies involving female athletes who were considered to be at an ‘elite’ level. For the purposes of this review ‘elite’ could refer to athletic performance at regional/county/state level; sport/country-specific measures and university/collegiate, international and/or national level; training; professionalism; involved in talent development and (3) studies that reported the *experiences* of symptoms of PFD and how this affects activities of daily living or sporting activity or quality of life (QOL). This information could be gleaned from questions included in a quantitative survey or qualitative interview-based research. Exclusion criteria were: (1) studies involving only recreational or leisure-time exercisers; (2) editorial opinion articles, letters and commentaries and (3) studies that did not report on the experiences of symptoms of PFD.

### Study selection and assessment of methodological quality

Two reviewers (ECQ and DD, ECQ and NF) independently screened studies using the inclusion/exclusion criteria based on titles, then abstracts and then full texts. Covidence software (Covidence systematic review software, Veritas Health Innovation, Melbourne, Australia. Available at www.covidence.org) was used for the process of screening and identified agreement between reviewers. Disagreement between two reviewers was resolved by consultation with a third reviewer.

Thomas et al. [20] devised a 12-point quality assessment criteria checklist that facilitated assessment of quantitative, qualitative and mixed methods studies. Subsequently, Panda et al. [21] developed a modified version of this checklist that was used to assess the methodological quality of this reviews included studies. Each criterion was scored ‘1’ if met and ‘0’ if not met, and three categories of methodological quality were identified: ‘weak’ (scores 0–6), ‘moderate’ (scores 7–9) and ‘strong’ (scores 10–12).

### Data extraction and data analysis

A double independent data extraction was conducted on the studies selected for inclusion (ECQ and DD, ECQ and NF).

Information was extracted and entered into a pre-designed extraction form in relation to the following study characteristics: authors; journal, year of study/publication, number of participants, participants’ characteristics, description of sport or athletic activity, methods of data collection, data collection instruments/tools, description of symptoms, methods of analysis and reporting of experiences concerning symptoms of PFD.

Information regarding athletes’ experiences of PFD was extracted from closed questions included in validated QOL instruments from closed questions and open-ended qualitative comments in questionnaires or from analysis of comments from focus group interviews.

There was insufficient qualitative information to carry out a meta-synthesis of the findings regarding the reporting of experiences. Thematic analysis occurred by organising the findings into themes that were tabulated and then further analysed [22]. Descriptive themes were developed. An iterative process was repeated until the themes were considered to be representative and answered the research question.

## Results

### Selection and quality of the studies

**Fig. 1.**
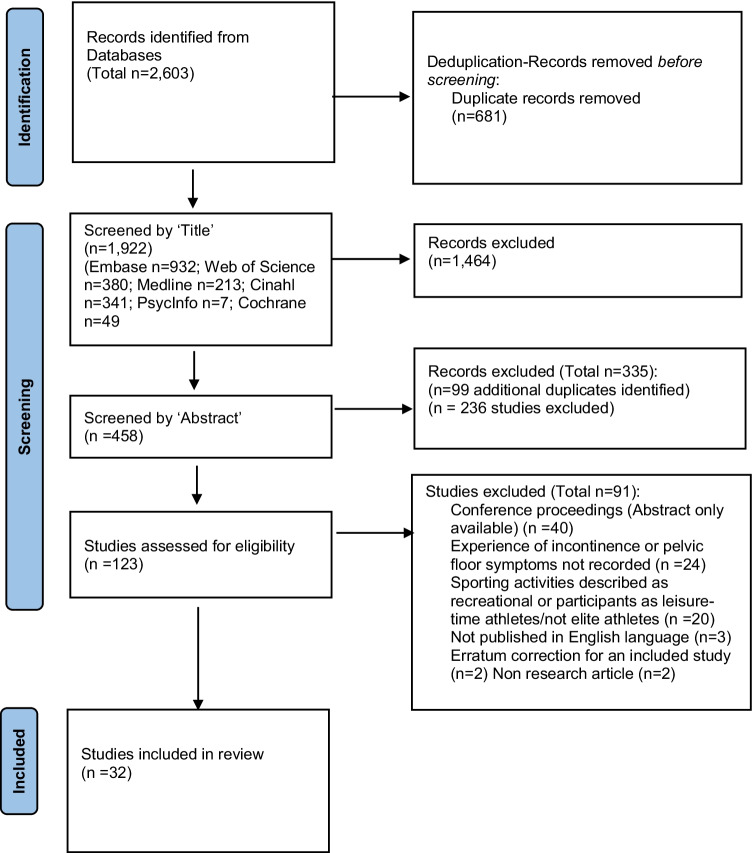
PRISMA flow diagram [18] for included studies

A total of 1922 studies remained after deduplication, 123 were screened at full-text and 32 studies met the inclusion criteria (Fig. 1). Of these, three studies were of weak quality [23–25], 28 studies were of moderate quality [8, 26–52] and one was of high quality [53].

### Descriptive analyses of the studies excluding information relating to the reporting of experiences

A summary of the study characteristics *excluding* the reporting of experiences is presented in Appendix 1. Studies were conducted in 14 countries and published between 1994 and 2021. Over half of the studies reporting on the experiences of PFD in the review were published within the last 5 years (56%, 18/32 studies) [8, 27–29, 32, 36, 38, 39, 42, 45–53]. Fifteen studies involved nulliparous participants [23, 24, 26, 27, 29, 31, 35, 37–39, 41, 42, 44, 45, 53] and 15 studies included both nulliparous and parous participants [8, 25, 28, 30, 32–34, 36, 40, 43, 46, 48, 50–52]. Two studies did not report information on parity of participants [47, 49].

Thirty-one of the 32 studies used a quantitative design and only one study by Jácome et al. used a mixed methods design (questionnaire and focus group) [40]. None of the studies in this review used a qualitative design only and there was considerable heterogeneity in study designs. Seven studies utilised purposively designed questionnaires [30, 33–35, 37, 43, 44] whilst the remaining 25 used or incorporated validated instruments. The most commonly used survey instrument was the International Consultation on Incontinence Questionnaire-Short Form (ICIQ-UI-SF), which was used in 13/32 (31%) of the studies [8, 26–29, 31, 32, 38, 39, 42, 46–48].

UI was the most commonly reported symptom of PFD and reported in all 32 of the studies. Twenty-two studies specifically described the prevalence of UI according to type, with SUI reported as the most prevalent in 21/22 studies [8, 23, 24, 26, 28–30, 32–36, 38, 40–43, 45–48]. Mixed urinary incontinence (MUI) was reported as the most prevalent form of UI in one study by Cardoso et al. [27]. Anorectal dysfunction (ARD) was reported in nine studies with the prevalence of constipation/straining to defecate reported in all of these studies [8, 26, 28, 29, 31, 32, 34, 46, 47] and anal incontinence (AI) reported in four studies [26, 29, 46, 47]. The prevalence of sexual dysfunction (SD) was reported in two studies [26, 29], the prevalence of POP was reported in four studies [26, 29, 41, 46] and the prevalence of pelvic pain was reported in two studies [24, 45].

### Experiences of pelvic floor dysfunction

Information regarding female athletes’ experiences of PFD was extracted from closed questions included in validated QOL instruments, from closed questions and open-ended qualitative comments in questionnaires and/or from analysis of comments from a focus group. The findings regarding the experiences of the athletes were grouped into five main themes (Table 1) and are summarised below. As previously mentioned, there was insufficient qualitative information to perform meta-synthesis of the findings.

**Table 1.** Athletes’ experiences of PFD: triggers reported for symptoms of PFD, strategies adopted by athletes to manage/mitigate/report symptoms of PFD and impact on QOL/daily life, performance and emotions (NR = not reported)

Study	Triggers for symptoms of PFD	Strategies adopted by athletes to manage/mitigate symptoms of PFD	Impact on QOL/daily life	Impact on performance	Impact on emotions
Almeida et al. 2016 [26]	**Type sport in competition or training-** UI- highest prevalence among the athletes who practiced artistic gymnastics and trampoline (88.9%) SUI-artistic gymnasts and trampolinists = 87%	48% (32/67) of athletes used strategies to avoid UI "**Emptying the bladder before training"** was the most reported strategy-31.4% (10/32)	NR	NR	NR
Cardoso et al. 2018 [27]	**Training-**UI in training in 61% (50/82) **Competition-** UI in competition 45% (37/82)	Strategies reported included: **Hydric restriction-**15% (12/82) **Use of pad-**12% (10/82) **Sought medical assistance-**4% (3/82) **Sought physiotherapeutic care-**0% **Mentioned UI to trainer-**0%	**Slight impact-**as measured by ICIQ-UI-SF, mean of 1.98 points for impact on QOL, reflecting a slight impact	**Did not affect sports performance** Most athletes considered UI did not affect their sports performance (no. % or n/n given)	NR
Carls 2007, USA [23]	**Type of movement/activity in sport-** sports 14%, exercises-11.6%, jumping-6.9% and weightlifting-2.3% **Coughing-**11.6%; **sneezing-**6.9% **Walking to bathroom with a strong urge-**11.6% **Hearing running water with urge**-4.7% (n/n unspecified)	**Avoided activities-**8% reported avoiding hobbies, social activities, sports,and exercises because of their SUI **Majority of athletes with UI did not speak to anyone about SUI**-92%	**Negative effect-**16% with UI reported a negative effect on their social life, sports or exercise	** NR**	NR
Carvalhais et al. 2017 [28]	**Training-**UI in training in 74.5% (82/110) **Middle/end of training sessions-**Of those reporting UI in training 84.1% (69/82) reported it happened at middle/end of training	14.6% (12/82) athletes with UI used strategies to ‘reduce visible leakage’ **Use of pad-**75% (9/12) of those that used strategies reported wearing pads	NR	**Affected sports performance**– 39.1% (32/82) considered that UI affected sports practice	NR
Carvalho et al. 2020 [29]	**Training sessions-**Of the cheerleaders who reported UI then 47.6% (10/21) reported UI during training	NR	NR	NR	NR
Caylet et al. 2006 [30]	**Training-**34.1% (15/44) reported UI in training **Second part of training sessions-**86.67% (13/15) reported that it happened only in the second part of training **Competition-**38.6% (17/44) reported UI **Second part of competitions-**58.8% (10/17) UI only in the second part of competition	**Majority of athletes did not speak to anyone about UI**-84% (37/44) **Spoke to trainer-**0% **Spoke to family doctor-**2.2% (1/44) **Spoke to sports doctor-**2.2% (1/44) **Spoke to family member-**11.3% (5/44)	NR	NR	NR
Da Roza et al. 2015 [31]	**Training-**UI in training-72.7% (16/22), all incontinent women stated that UL started only *after* they began trampoline training **Higher ranking in sport-** a positive association observed between higher ranking in the national championship and ICIQ score (r = 0.573, *p* = 0.05)	NR	Self-reported overall impact of UI on QOL was reported as **“Not interfere”** or “**Interfering “mildly”** by 68.8% (11/16) of the incontinent athletes **Affected daily life-**Only 1 athlete reported that loss of urine had a great effect in her daily life	NR	**Embarrassment-**athletes with higher training volumes felt more embarrassment and discomfort with urine leakage (no % or n/n given)
Dobrowolski et al. 2020 [32]	**Competition and Training** (% and *n* = unclear**)** **Type of movement/activity in sport-**SUI occurred most often among these athletes when performing “double unders” 67% (36/54) and “triple unders” 86% (48/56)	***Strategies used by retired RS with UI during RS (survey 2)*** **Voiding before events**-72% (46/64) **Voiding between events**-71% (45/63) **Using containment, e.g. pads or tampons-**38% (24/63) **Limiting fluid intake-**20% (12/61) **Sought treatment for UI**-0% Only one female athlete identified that SUI was one of eight reasons for retiring from RS	**Slight impact of UI** on QOL-median ICIQ-UI-SF score in incontinent athletes was 4 (IQR 3–6), indicating a slight impact of UI on their overall QOL	**Affected sports performance**-some athletes stopped participating in “double unders” and “triple unders” events because of SUI Attrition from participation in ‘double unders’ and ‘triple unders’ due to SUI was 6% in competition; 16% in practice	NR
Dockter et al. 2007 [33]	**Coughing, sneezing and/or laughing-** 16.5% (*n* = 18/109) reported ‘sometimes’ or ‘frequently’ **During physical exertion (lifting, running, jumping, abrupt movements)** 16.5% (*n* = 18/109) reported ‘sometimes’, ‘often’ or ‘always’ **UI urge to void (problem on way to toilet)-**10.1% (11/109)	**Prevention strategies used by-**38.53% (42/109) ***Strategies*** **Increase frequency of urination/go before event/run to bathroom-**42.85% (18/42) **Holding urine or avoiding laughing-**16.67% (7/42) **Doing nothing to prevent leakage-**30.95% (13/42) **Other (not defined)-**9.52% (4/42)	NR	NR	NR
Eliasson et al. 2002 [35]	**End of the exercise session-**Leakage during jumping (answered by 21 participants mainly: “at end of exercise session” 47.6% (*n* = 10/21), **New, strenuous and difficult exercises-**reported as triggers 38.1% (*n* = 8/21) **Type of activity in sport-** UI reported occurring ‘in double somersaults” 23.8% (*n* = 5/21) Women in leakage group had been training longer (*p* < 0.04) and more frequently(*p* < 0.03)	***Strategies used by those with UI*** **Protective pads-**82.1% (23/28 with 12/23 of these always) **Frequent toilet visits-**(n/n unspecified) **Limiting fluid intake-**(n/n unspecified)	NR	NR	**Embarrassment-** 51.4% (18/35) athletes reported that they were embarrassed and considered the leakage a social or hygienic problem (61.1%, 11/18-very embarrassed”)
Eliasson et al. 2008 [34]	**Type of activity in sport-** 28% (39/138) of athletes connected leakage with specific exercise- high jumps or somersaults especially double ones **Training-** athletes reported UI occurred more often during training (*p* = 0.022) **Coughing, laughing- (**numbers not specified); **on way to the toilet- (**numbers not specified); **during other physical activities-** (n/n unspecified)	**Protective pads-** 78 % (45/58) competitive athletes (CG) used sanitary pads, and this was significantly more than recreational group (RG) 43 % (34/80) (*p* < 0.001) **Discussed their leakage-** 72% (*n* = 99/138) of athletes (Competition Group & Recreation Group) had discussed their leakage with someone, most of them with friends, team coach or parents	**Affected daily life-***when experiencing UI during trampolining-*36% (20/85) competitive athletes were affected in their daily life 61% (31/85) were affected psychologically	**Affected sports performance-** 12%, (*n* = 16/133) of athletes (Competition Group & Recreation Group) had stopped trampolining due to the leakage	NR
Faulks & Catto 2021, [36]	**Type of activity in sport-** Being tackled- 67% (26/39) Sprinting- 61% (24/39) Jumping 54%- (21/39) Making a tackle- 49% (19/39) Changing direction while running-39% (15/39) Jogging-33% (13/39) Grappling or wrestling-31% (12/39) Scrummaging- 28% (11/39) Lifting- 23% (9/39) Throwing-21% (8/39) Catching- 18% (7/39)	NR	NR	**Affected sports performance-** 28% (11/39) reported effect of SUI on performance during game or training setting 5% (2/39) reported SUI as barrier to playing rugby union in the future	NR
Ferreira et al. 2014 [37]	**Training -** experimental group/EG-87.5% (14/16), Control Group/CG-81.3% (13/16) **Competition-** EG-12.5% (2/16), CG-18.8% (3/16)	**Protective pads-** wearing pads EG, 68.8% & CG, 68.8%, (11/16) **Voiding before sports-** going to the bathroom prior to sport (EG, 87.5%,14/16; CG, 100% 16/16) **Reducing liquids-** reducing fluids prior to sport (EG, 56.3%, 9/16/; CG, 50.0%, 8/16)	NR	NR	NR
Gram & Bo-2020 [38]	34 athletes reported UI and 21 athletes SUI Of the 21 with SUI then: **Physical Activity (PA)-** UI on PA- 57.1% (12/21) **Coughing & sneezing-** UI in 9.5% (2/21)	NR	**Mild interference on QOL-**Mean score ICIQ-UI-SF UI interfering with daily life was 1.2 (SD 1.1)	**Affected sports performance-** UI affected sports performance- 70.6% (24/34) gymnasts with UI reporting that the condition had some affect	**Fear-** Of the 34 gymnasts with UI, 29.4% (10/34) reported to be afraid of visible leakage and 14.7% (5/34) that leakage would happen again
Hagovska et al. 2018 [39]	**Physical activity (PA)-** slight UI on PA- in 6.14% sportswomen (n/n unspecified)	NR	**Significant negative effect on QOL-** Significant negative correlation was observed between SUI and the overall I-Qol questionnaire score (r = 0.522, *p* < 0.001)	**Affected sports performance-** Significantly worse parameters were recorded in the group of sportswomen (r = 0.648, *p* < 0.001) in the I-QoL in sub-scales ‘avoidance & limiting behaviour score’	**Embarrassment-** Significantly worse parameters recorded in the sportswomen in the I-QoL in sub-scales- psychosocial impact score & social embarrassment score (*p* < 0.001)
Jácome et al. 2011 [40]	**1.Questionnaire** 41.5% (44/106) **Urgent need to go to the bathroom-** 43.1%, (19/44) **Coughing-**38.6%, (17/44) **Practicing sport-** 36.4%, (16/44) **Sneezing-** 31.8%, (14/44) **Laughing-** 31.8%, (14/44) **2. Focus group** **Activities requiring physical effort** **Jumping**	**1.Questionnaire** **Discussed their leakage-**38.6% 20.4% (9/44) discussed it with a friend, 11.4% (5/44) with a relative, 4.5% (2/44) with a health professional 2.3% with the team coach (1/44) **2. Focus group** *strategies used to limit UI* **Restriction of liquids, preventative urination** **Performance of physical activities in restricted way, sought help-**no athlete in the focus group had sought help from a health professional for UI	**2. Focus group (n = 7)** Despite their concerns about UI, the athletes stated that the condition had no current impact on their daily lives	**2. Focus group** **Affected sports performance-** Resulted in ‘performance of physical activities in a restricted way’	**2. Focus group** *When urine loss occurred, subjects reported being:* **Concerned** **Annoyed** **Frustrated** **Fearful** ‘that a new activity might trigger another leakage’ (n/n unspecified)
Larsen & Yavorek 2006 [41]	NR	**Use of pad-** only one woman reported using pads due to UI 3.6% (1/28)	None of the women felt that urine loss constituted a problem	NR	NR
Ludviksdottir et al. 2018 [42]	**Coughing & sneezing** (n/n unspecified)	NR	11/18 athletes with UI **Disturbed daily life-** scale 0-10 36.4 % (4/11) no disturbance 63.6% (7/11) score ≤ 5.	NR	NR
Nygaard et al. 1994 [44]	**Practice-UI** 16% (23/144) **Competition-**16% (23/144) **Type of movement/activity in sport-** Jumping/legs apart (30%), Jumping with legs together (28%), Running (30%), Impact on floor during dismount/after flips 14%). ***Daily Activities (excluded leaking ‘rarely’)*** **Coughing-** 15%, **sneezing-** 6%, **heavy lifting-** 3%, **walking to the bathroom-** 29%, **sleeping-** 6%, **on hearing running water-** 11%	**Use of pad-** Only one woman stated that she wore a pad because of the urine loss **Discussed their leakage-**(n/n unspecified) Almost half of the athletes discussed the incontinence with a teammate, < 5% had discussed the UI with a trainer, coach, physician, nurse or family member	NR	NR	**Embarrassment-** 38% of the athletes felt embarrassed **Anxiety** 22% describing anxiety, **fear-**6% expressed fear about the condition (n/n unspecified)
Nygaard 1997 [43]	**During Olympic sport** **High impact-** 35.8% (19/53) **Low impact-** 4.5% (12/44)	**Strategies during Olympic activity not recorded** **Current strategies-Use of pad (currently)-**3 of the high-impact athletes and 1 of the low-impact athletes wearing a pad daily (n/n unspecified **Discussed their leakage-**4 athletes sought medical treatment for UI (n/n unspecified)	NR	**Affected sports performance (currently)-** athletes stopped an activity because of UI (n/n unspecified)	NR
Pires et al. 2020 [53]	**Coughing sneezing and running (as per KHQ Part II Q5)-** 69.2% (9/16) of all athletes (both CG and EG) CG-Control group EG- Experimental group	NR	**High QOL in participants-** Pre-intervention the mean global score in the KHQ was low in both groups (CG: 8.80 ± 4.62; EG: 6.35 ± 5.19 in EG) indicating high QoL	**Affecting physical activities (PAs) (pre-intervention) -**69.2%, (9/13), responded ‘a little’, 7.7% (1/13) ‘moderately’ re bladder problem affecting PAs	NR
Poswiata et al. 2014 [24]	NR	NR	**Scale from 0–100, the degree to which the respondents found the UI symptoms bothersome**- Not bothered -29.46% Slightly bothered 42.86% Moderately bothered -18.75% Significantly bothered 8.04% Heavily bothered- 0.89% (n/n unspecified)	NR	NR
Rodríguez-López, 2021 [8]	**Type of movement/activity in sport** Trigger for leakage while training: Lifting weights- 9% (14/156) Running 19.2% (30/156) After running 4.5% (7/156) Jumping 43.6% (68/156) After jumping 2.6% (4/156) Trunk rotation 1.3% (2/156) Forward flexion 0.6% (1/156) **Days spent training:** In female athletes, weak correlation between UI and days of training/week (r = 0.104; *p* = 0.028)	NR	**Severity of condition:** ISI scores reported indicated that among female athletes with UI (n = 168): 3% (5/168) described condition as severe 28.6% (48/168) as moderate 68.5% (115/168) as slight	NR	NR
Sandwith & Robert 2021 [45]	**Competition and training** Rugby game competition- 90% (46/51) **Type of movement/activity in sport** Tackled/hit- 88% (45/51) Running- 41% (20/51) Weight training- 18% (9/51) **Time spent training** Athletes who leaked urine reported more hours of training/week (*p* = 0.008). For every additional hour of training, the risk of UI increased by 15.3% (2.9%–29.3%, 95% CI)	**Use of pad-** None of the athletes disclosed use of any incontinence products or pads during exercise **Discussed their leakage/Sought treatment-** Only one athlete had discussed her UI with a health professional None of players had received any treatment for UI Several players 18% (9/51) were interested in receiving treatment for their urinary incontinence	**Degree of bother associated with UI** % Of players who reported that UI was ‘not a problem/only a small problem’: Rugby game competition- 100% (46/46) Tackled/hit- 98% (44/45) Running- 41% (20/20) Weight training- 100% (9/9)	NR	NR
Skaug et al. 2020 [46]	**Triggers for UI** **Type of movement/activity in sport (n = 82)** Heavy lifting (1-5RM)- 78% (64/82) Deadlift- 63% (52/82) Squat- 56% (46/82) Weightlifting with belt- 34% (28/82) Clean lift- 13% (11/82) Weightlifting (>6 reps)- 12% (10/82) Power/explosive training- 12% (10/82) Bench Press- 2% (2/82), Snatch lift- 1% (1/82) **Training& Competition- -** Most women with SUI reported UI during training- 91.5% (75/82) and more than half during competition 56.1% (46/82) **Body mass index was** the only factor found to have a significant positive association with SUI **Triggers for AI** **Training and competition-** Gas AI- 89.1% (123/138) experienced leakage during training or competition Liquid AI- 23.8% (14/59) Solid AI- 15.4% (2/13) **Level of competition** international level of competition was positively associated with AI Time spent training: AI-weightlifting training of <4 days per week had a significant negative association	**Strategies to manage UI (n/82)** **Use of pad-** 54.9% (45/82) reported the use of pads to protect against visible leakage and 7.3% (6/82) used intravaginal tampon **Voiding before sports activity-** 86.6 (71/82) voiding before training or competition **Restriction of fluids-** 13.4% (11/82) decreased fluid intake **Performance of PA in restricted way-** 19.5% (16/82) reported they would occasionally avoid training or specific exercises because of UL **Discussed their leakage/sought treatment-** 25.6% (21/82) had never spoken about the condition with anyone **Pelvic floor muscle training (PFMT)-** 42.8% (77/180) women did not know *why* and 44.4% how (80/180) to train the PFM 78.3% (141/180) women responded they would do PFM training to prevent or treat PFD if they knew how	**Degree of bother associated with UI** ICIQ-UI-SF -Mean impact of UI on daily activities was 1.8 (SD: 2.0, range: 0–9), with 11 (12.2%) scoring ≥ 5 **Degree of bother associated with AI** Of women reporting AI, the mean bother of accidental loss of gas, liquid and solid stool was 2.3 (SD: 2.5, range: 0–9), 2.0 (SD: 2.5, range: 0–9) and 2.2 (SD: 2.8, range: 0–9), respectively The percentage of women scoring ≥ 5 on bother was Gas AI- 15.9% (22/138) Liquid stool- 15.3% (9/59) Solid stool- 15.4% (2/13)	**Affected sports performance –** 87.8% (72/82)) of those with SUI reported a negative effect of UI on sports performance	**Impact of SUI** **Loss of concentration-** 51% (42/82) **Fear of visible leaking-** 59% (48/82) **Fear of Urine odour-** 34% (28/82) **Embarrassment-** 33% (27/82) **Negative effect on performance-** 27% (22/82) **Feeling frustrated, annoyed or worried-** 24% (20/82) **Fear of leakage happening-** 23% (18/82) **Making more mistakes -**13% (11/82)
Skaug et al. 2022 [47]	**Triggers for UI** **Type sport -**Proportion of SUI was significantly lower in cheerleaders compared to artistic gymnasts (and team gymnasts (*p* < 0.001) **Type of movement/activity in sport (n = 210)** Running- 4% (8/201) Jumping- 50% (101/201) Take-off to a gymnastic or acrobatic element- 67% (135/201) Land from a gymnastic or acrobatic element- 60% (121/201) In air during a gymnastic or acrobatic element- 13% (26/201) Trampoline or trampolette- 51% (103/201) **Training& Competition-** Most athletes with SUI reported leakage during training – 98% (198/201) 44.8% during competition (90/201) **Triggers for AI** Years with specialization in gymnastics/cheerleading was the only variable found to be positively associated with AI **Training and competition-** Gas- 87.6% (227/259) experienced leakage during training and/or competition: 38.2% (99/259) rarely, 35.1% (91/259) occasionally, 12% (31/259) often and 2.3% (6/259) all the time Liquid AI- 22.3% (29/130) experienced leakage during training and/or competition; 18.5% (24/130) rarely and 3.8% (5/130) occasionally Solid AI- 17.9% (7/39) reported leakage during training/competition, all experienced it rarely	**Strategies to manage UI (n/201)** **Use of pad-** 28.4% (57/201) reported use of pads to protect against visible leakage and 4.5% (9/201) used intravaginal tampon **Voiding before sports activity-** 66.7% (134/201) prevoiding training/competition **Restriction of fluids-** 8.5% (17/201) decreased fluid intake **Performance of PA in restricted way-** 22.4% (45/201) reported they would occasionally avoid training or specific exercises because of UL **Discussed their leakage/sought treatment-** 26.4% (53/201) had never spoken about UI 13 (6.5%) had spoken with their coach and 12 (6.0%) with health care personnel, 115(57.2%) had spoken about UI with their teammates, 76 (37.8%) with friends and 40 (19.9%) with a parent **Pelvic floor muscle training (PFMT)-** 0.9% (3/319) reported they did or had tried PFMT 41.4% (132/319) of the athletes had never heard about the PFM. 73.7% (230/319) women responded they would do PFMT to prevent or treat PFD if they knew how 12.2% (39/319) of the athletes reported that they had heard about the PFM from their coach, 10% (32/319) (from teammates, 19.1% (61/319) from health personnel and 16.9% (54/319) from other sources (friends, siblings or parents) The mean self-rated knowledge of the PFM was 1.5 (SD: 1.7) of 10. Thirty-two (10.0%) knew how and 58 (18.2%) why to train the PFM	**Impact on daily life associated with UI** ICIQ-UI-SF score mean impact of UI on daily activities was 2.5 (SD: 2.4, range: 0–10), with 46 (21.4%) scoring ≥ 5 **AI** Of females reporting AI, mean bother of accidental loss of gas, liquid and solid stool was 3.0 (SD: 2.6, range: 0–10), 2.3 (SD: 2.3, 0–10) and 2.4 (SD: 2.4, range The number of athletes scoring ≥ 5 on bother 0–10) was respectively: Gas AI – 26.6% (69/259) Liquid stool- 15.4% (20/130) Solid stool- 15.4% (6/39)	**Affected sports performance –** 82.6% (166/201) of those with SUI reported a negative effect of UI on sports performance	**Impact of SUI** **Fear of visible leaking-** 66% (133/201) **Embarrassment-** 65% (131/201) **Fear of Urine odour-** 51% (103/201) **Fear of leakage happening-** 39% (78/201) **Loss of concentration-** 31% (62/201) **Feeling frustrated, annoyed or worried-** 29% (58/201) **Negative effect on performance-** 18% (9/201) **Making more mistakes -**11% (22/201) **Impact of SUI** **Fear of bowel leakage happening-** 49% (13/) reported that they sometimes or more often were worried about bowel leakage
Thyssen et al. 2002 [25]	**Training and competition-** UI in training- 95.2% (119/125) UI during competition- 51.2% (64/125)	**Use of pad-** 60.2% (91/151) occasionally wore a pad/shield **Restriction of fluids-** 6.6% (10/151) reduced liquid intake to reduce UL **Discussed their leakage/sought treatment-** Only 3.3% (5/151) discussed UI with their doctor **Pelvic floor muscle training (PFMT)-** 4.6% (6/151) had completed a pelvic floor training program because of UI	33.8% (51/151) considered the UL as a problem 21.1% (32/151) as a hygienic problem	NR	NR
Velázquez-Saornil et al. 2021 [48]	**Training and competition-** Leakage caused by exercise or physical exertion accounts for 64.3% **Type of sport/athletics- (**No significant relationship UI/sporting discipline) Greatest number of athletes with UI practice long-distance running, represented by 32.1% of all women experiencing UI Jumping events lowest percentage 10.7% UI	**Use of pad-** 58.6% (17/28) of women use protection 39.3% (11/28) wet their underwear	0% of athletes considered that UI affected their daily life	46.4% (13/28) were affected in their sporting environment	**Anxiety/depression-** 14.3% (4/28)
Wikander et al. 2019 [49]	**UI- Training and competition lifts** (n/n unspecified) ***Voluntary comments by 27 women*** ***Type of activity in sport-*Deadlifts-** 40.7% (n = 11), **squats-** 18.51% (5/27) **front squats-** 7.4% (2/27) **Wearing a belt lifting-** 18.51% (5/27) **End of sets-**14.8% (4/27) UI moderate to heavy weights **Heavier weights-**51.9% (14/27) UI with very heavy/maximal weights ***Activity outside sport-*Jumping-** 14.8% (4/27) **Sneezin**g- 7.4% (2/27)	**Strategies to control or minimise UI** **Pelvic floor exercises-** 7.4% (2/27) **Not wearing a belt-** 7.4% (2/27) **Prophylactic voiding-** 11.1% (3/27) **Improving their diet** 3.7% (1/27)	NR	NR	NR
Wikander et al. 2020, [50]	**Training and competition-** UI during competition 32.1%, (145/452) Training 37.4% (169/452) 38.3% (n = 173/452) experienced UI during training and/or competition *as well as* daily life 17.7% (80/452) experienced UI during training and/or competition but *NOT* in daily life **Type of movement/activity in sport-** High impact high repetition activities involving jumping and running activities most likely to cause UI - Jumping rope 39.16% (177/452), Double-unders 36.95% (167/452), Trampoline 25.00% (113/452), Running/Jogging 20.57% (93/452) Activities least likely to provoke UI were low impact, body weight activities such as lunges UI during high repetition sets- 28.7% (N = 60/208) **End of session** 50% (30/60) indicated that UI was most likely to occur at the end of high repetition sets **Intensity-** 28.2% (N = 59/208) of incontinent women reporting UI during heavy sets	**Pelvic floor muscle training (PFMT)-** 73.6% (n = 153/208) of participants who had experienced UI at some point had never undergone a pelvic floor assessment. 26% (n = 54/208) of women who reported UI at some point in their life were not confident in their ability to correctly perform pelvic floor exercises	NR	NR	NR
Wikander et al. 2021A [51]	**Training and competition-** 23.1% (111/480) had experienced ‘athletic incontinence’ 17.9% of women (86/480) had been continent before commencing powerlifting now UI in training or competition but not during everyday activities (Type 1 athletic incontinence) 5.2% (25/480) had UI before commencing powerlifting but are now continent during everyday activities while continuing to experience UI during training or competition (Type 2 athletic incontinence) **Type of movement/activity in sport-** 30.6% (147/480) experienced UI in competition during maximum lift attempts 40.4% (194/480) experienced UI in training during maximum lift attempts 12.5% (60/480) during sumo deadlifts 35.2% (169/480) during high repetition sets **End of session-** 64.5% (109/169) stated UI worse at end of sets **Intensity-** 79.3% (N = 134/169) indicated that UI was only an issue if the sets were heavy	**Strategies to control or minimise UI were listed under the following headings (no. N = or % given):** ***Bracing related-*** Modifying technique, ± belt use, ± PFEs ***Preparation/setup related-*** Blowing/exhaling prior to lift, pelvic floor lift, dynamic warmup/post-workout stretching routine ***Pelvic floor related-*** PFMT, pre-contraction of PF ***Technique/form/breathing related-*** exhaling during lift, ribcage positioning, bracing PFMs ***Other training related-*** Avoid wearing belt, **frequent voiding, fluid restriction,** take spare underwear to gym and competitions ***General-*** **wear protection**, physiotherapy treatment/PFMT/stretching/relaxation and massage, TENS, core exercises, avoid straining/certain activities/laughing, reducing caffeine ** Sought treatment-** 20.9% (49/234) with UI had undergone a pelvic floor assessment **Pelvic floor muscle training (PFMT)-** 71.71% (344/480) of participants stated that they were either confident/very confident re PFEs	NR	NR	NR
Wikander et al. 2021B [52]	**Training and competition-** 16.2% (31/191) of weightlifters reported ‘type one athletic incontinence’ 17.8%, (34/191) UI in competition i 25.7%, (49/191) UI in training 25.7%, (49/191) **Type of movement/activity in sport-** 57.1% (40/70) experienced urinary leakage during high repetition sets Max. effort lift in competition 16.8% (32/191) Max. effort lift in training 24.6%, (47/191) Wearing a belt provoked UI n 3.7% (7/191) **Intensity-** 67.5% (27/40) of these women indicated that leakage was only an issue if the sets were heavy **End of session-** 50% (20/40) who experienced UI during high repetition sets stated that the leakage was more likely to occur at the end of the set	**Strategies to control/minimise UI (no. N =, % given)** Antibiotics for recurring urinary tract infections, yoga and Pilates, **emptying bladder before training and frequent urination during training sessions/competitions** **Engaging pelvic floor before lifting** Focusing on breathing, bracing core before lifts, trying to not over brace, **wearing a pad**, using a tampon or avoiding the use of tampons, **practicing pelvic floor exercises outside training**, release work/massage, focus on pelvic mobility, core training, not overtightening belt, wearing dark-coloured clothing, maintain a low body mass, crossing legs before sneezing **Sought treatment-** 24.3% (17/70) of those with UI had undergone a pelvic floor assessment **Pelvic floor muscle training (PFMT)-** 77.1% (54/70) of those with UI stated that they were confident/very confident in their ability to perform pelvic floor exercises	NR	NR	NR

Key findings highlighted in bold

#### Theme 1: Triggers for symptoms of PFD

The first theme *Triggers for symptoms of PFD* was reported in 30/32 (94%) of the studies. ‘Competition, training and physical activity’ was the most common trigger reported in 26/30 studies [8, 25–32, 34, 35, 37–40, 43–52]. In 7/26 studies [28, 30, 35, 49–52], the PFD occurred at the end/latter part of the competition or training session. ‘Specific movements during activity (sporting and daily life)’ was the next most common trigger reported in 16/30 studies [8, 23, 32–36, 40, 44–47, 49–52]. Being ‘on the way to toilet/sudden need to go to toilet’ was reported as a trigger in 5/30 studies [23, 33, 34, 40, 44] suggesting symptoms of mixed urinary incontinence (MUI).

#### Theme 2: Strategies adopted by athletes to manage/mitigate symptoms of PFD

The second theme *Strategies adopted by athletes to manage/mitigate symptoms of PFD* was reported in 23/32 (72%) of the studies. The ‘use of pads/containment strategies’ was the most commonly identified strategy reported in 16/23 studies [25, 27, 28, 32, 34, 35, 37, 41, 43–48, 51, 52]. Fourteen of the 23 studies found that athletes ‘Discussed condition with others/sought help’. However, very few participants discussed/sought help for their PFD with a health professional [23, 25, 27, 30, 32, 34, 40, 43–47, 51, 52]. ‘Increased frequency of urination/pre-voiding/voiding during events’ were strategies adopted by athletes in 10/23 studies [26, 32, 33, 35, 37, 40, 46, 47, 49, 52] and ‘fluid restriction’ was reported in 9/23 studies [25, 27, 32, 35, 37, 40, 46, 47, 51]. ‘Modification or avoidance of certain activities/movements’ was reported in 7/23 studies [23, 40, 46, 47, 49, 51, 52]. Pelvic floor muscle exercises/training (PFMEs/PFMT) was a strategy used by participants in 7/23 studies [25, 46, 47, 49–52].

#### Theme 3: Impact of PFD on QOL/daily life

The third theme *Impact on QOL/daily life* was reported on in 18/32 (56%) of the studies. The findings were diverse. Hagovska et al. [39] described a significant negative correlation between athletes’ prevalence of SUI and overall I-Qol score, and Eliasson et al. [34] reported that, as a result of their PFD, 36% (20/85) of the trampolinists were affected in their daily lives and 61% (31/85) were affected psychologically. However, the majority of the studies (16/18) reported that, for most of the participants, PFD did not have a marked impact on the athletes’ QOL/daily life [8, 23–25, 27, 31, 32, 38, 40–42, 45–48, 53].

#### Theme 4: Impact on performance

The fourth theme *Impact on performance* was reported on in 12/32 (38%) of the studies. One study reported that PFD ‘did not impact performance’ [27]. The remaining 11/12 studies reported that PFD had an ‘effect on some of the athletes’ performance’ [28, 32, 34, 36, 38–40, 46–48, 53] and 6 of these studies reported that some athletes had stopped an activity or limited their behaviour/activity in their sport [32, 34, 36, 39, 40, 43].

#### Theme 5: Impact on emotions

The fifth and final theme *Impact on emotions* was reported in 9/32 (28%) studies. The following negative emotions in some athletes were reported as a consequence of PFD: ‘embarrassment’ in 6/9 studies [31, 35, 39, 44, 46, 47]; ‘fear’ in 5/9 studies [38, 40, 44, 46, 47]; ‘concern/anxiety/worry’ in 5/9 studies [40, 44, 46–48]; ‘annoyance’ in 4/9 studies [40, 46–48]; finally, ‘frustration’ was an emotion reported by some athletes in in 3/9 studies [40, 46, 47].

## Discussion

The primary aim of this review was to investigate the experiences of symptoms of PFD in elite female athletes. There was heterogeneity in study designs including a wide variety of athletic/sporting activity and most of the information regarding the athletes’ experiences came from quantitative research studies where the athletes’ experience of PFD was not the main focus. This was predominantly quantitative research involving questionnaires. Only one mixed methods study by Jacome et al. [40] included a qualitative component to elicit athletes’ experiences and this involved a relatively small focus group (*n* = 7). It is notable that > 50% of the studies were published within the last 5 years and this may indicate an increased interest in understanding the impact of PFD on athletes. Five main themes were identified and only three studies contributed findings to all five themes. Two of these were recent studies by Skaug et al., which, in addition to investigating prevalence, also investigated the impact and bother of PFD on powerlifters and weightlifters [46] and cheerleaders and gymnasts [47]. The third study was the mixed methods study by Jacome et al. [40].

Systematic reviews regarding female athletes and PFD to date have predominantly focused on the prevalence of UI in female athletes [9–13]. As prevalence was not the main focus of the current review, the prevalence reported here was only that relating to those studies reporting elite athletes’ *experiences* of PFD and does not allow direct comparisons with previous reviews regarding prevalence of PFD in athletes.

The fact that UI was reported in all the studies in this review was not surprising as UI is the most common form of PFD [54]. Similar to reviews by de Mattos Lourenco et al. [10] and Rebullido et al. [14], the ICIQ-UI-SF was found to be the most commonly utilised survey instrument. The prevalence and experiences of other forms of PFD including POP, ARD, sexual dysfunction and pelvic pain reported in the studies were also identified in this current review and it is notable that in all previous systematic reviews concerning athletes to date only the prevalence of UI was analysed. It is suggested that future systematic reviews should consider including the prevalence of other types of PFD to provide a more complete picture of PFD in these athletes. Bø and Nygaard [16] in a narrative review concerning physical activity and PFM function, identified AI and POP as understudied research outcomes.

This current review included only studies involving ‘elite’ athletes. Swann et al. [55], in a systematic review that aimed to evaluate how sports psychology research has defined elite athletes, identified eight broad categories of an ‘elite or expert athlete’. These included experience, international and/or national level, training, professionalism, involved in talent development, regional level, sport/country-specific measures and university. Williams et al. [56] suggested that for team sports, the recommendation for defining ‘elite' is that success in highly competitive leagues and competitive experience should be given priority over international experience. In this current review we used a definition of ‘elite’ based on the recommendations from the above literature. It remains difficult, however, to find a consistent definition of the term ‘elite’ as it refers to athletes. Almousa and Bandin Van Loon [9], in a systematic review investigating the prevalence of UI in nulliparous female athletes, highlighted that many studies failed to use level of sports (professional, amateur) to classify the participants.

The fact that ‘competition, training and physical activity’ was most commonly reported as a trigger for symptoms of PFD followed by ‘specific movements during activity (sporting and daily life)’ may not be surprising. Such activity may cause an increase in intra-abdominal pressure, impact the pelvic floor and is associated with SUI [1], the most prevalent type of PFD reported in this review. However, questions surrounding the effect of physical activity and exercise on the pelvic floor remain unresolved in the research literature and a need for further high-quality research to fill the gaps in knowledge concerning the role of strenuous physical activity in symptoms of PFD has been identified [16]. It is also be suggested that that future research regarding triggers for symptoms of PFD and when the symptoms occur in female athletes should be conducted as this may help inform the design of a pathway pelvic floor muscle (PFM) rehabilitation programme for such athletes.

In seven of the studies the PFD was reported as presenting in the second half/latter part of training or competition, and this may point to PFM fatigue. Previous research has suggested that, while some uncertainty remains regarding the extent to which PFM fatigue affects UI, the development and/or worsening of UI may be influenced by PFM fatigue [57–59].

In this review the studies that reported on strategies adopted by athletes to manage/mitigate symptoms of PFD indicated that, whilst many athletes reported wearing pads, pre-voiding and restricting fluids, few appeared to seek attention from a health professional. Similarly, de Mattos Lourenco et al. [10] also reported strategies to prevent UI including use of pads, pre-voiding and limiting fluid intake and suggested that reasons why athletes do not discuss the condition or seek help may include the fact that they feel ashamed or perceived that it is normal or inevitable at their age. Another possible reason may be that the athletes consider that their use of some strategies such as pre-voiding may be sufficient to manage their UI. Interview-based, qualitative research may assist in investigating this issue further.

UI is a treatable condition. Recent research has shown that PFMT in young continent women resulted in improved muscle activation and pelvic floor muscle strength [60]. Findings of a Cochrane review [61] have shown that PFMT can cure or improve all types of UI but is most effective in SUI. Similar to the findings of other reviews, we suggest that it is important to provide education resources for athletes regarding symptoms and treatment options for PFD and educate the professionals (health professionals and coaches/trainers) involved with these athletes about the prevalence of PFD in athletes and increase screening for symptoms [9, 10, 12, 13].

In this current review the findings regarding the impact of PFD on female athletes’ QOL/daily life were quite varied and consequently no conclusion can be reached. Future research could include systematic reviews of UI and QOL in athletes and conducting qualitative research may deepen knowledge and understanding of the impact and experiences of PFD on female athletes’ QOL and daily life. UI has been shown to have a considerable impact on women’s lives [6] and have an adverse effect on QOL [3, 5]. A systematic review and meta-analysis on UI and QOL in both sexes reported that UI was associated with poor QOL, but an acknowledged limitation of the review was that only cross-sectional and case-control studies were included [4].

The fact that 11/12 studies reporting on the impact of PFD on performance reported that it had an effect and, that some athletes had stopped or limited their activity during their sport, is of concern. A need for further research to investigate the mechanisms causing PFD in athletes has been identified [38]. It would also be interesting to evaluate if athletes who limit their sporting activity because of PFD have considered seeking or have sought treatment for their symptoms. Further mixed-methods or qualitative, interview-based research may assist in investigating this issue in athletes.

Only nine (28%) studies in this review reported on the impact of PFD on the athletes’ emotions and there was limited information given in some of these studies. Embarrassment was the most frequently reported emotion followed by fear, concern and anxiety. It is important that athletes are educated regarding PFD and the treatment options available to help allay, mitigate and resolve some of these negative emotions surrounding UI. Mendes et al. [6] reported that UI is a condition that can involve embarrassment, stigma and distress in women. Once again, we suggest that further qualitative, interview-based research may assist in investigating the impact of PFD on the athletes’ emotions.

## Limitations

Limitations of this review included the heterogeneity of the study designs, the inclusion of studies published in the English language only and that the prevalence reported in this review only referred to the prevalence of PFD in studies that reported on female athletes’ experiences of PFD. However, the main limitation of this review was the paucity of qualitative evidence that was available in the research literature concerning athletes’ experiences of PFD.

## Conclusion

This review explored the experiences and impact of PFD in elite athletes and found a relative paucity of qualitative evidence. Five main themes were identified. Further qualitative and mixed methods, sports-specific research may serve to deepen knowledge and understanding of elite female athletes’ experiences and impact of PFD on athletes’ sporting activities and their daily lives and enrich the understanding of this condition in women competing at an elite level.

Recommendations for qualitative research include further investigating:

- The impact of PFD on elite female athletes’ emotions
- Why do many elite female athletes not discuss their PFD with others?
- Triggers for PFD reported by elite female athletes
- Strategies adopted by elite female athletes to manage/mitigate their PFD
- Why do many elite female athletes not seek help from a health professional?
- Do elite female athletes who limit their sporting activity due to PFD seek treatment?

## Appendix 1

Table 2

**Table 2.** Summary of characteristics of the included studies excluding information regarding experiences

Author/year/country	Aim	Study Design	Sample size (n)	Participants/Type of sport	Age of participants	Data Collection*	Analysis	Prevalence of PFD symptoms** in athletes (a. UI; b. ARD; c. SD; d. POP; e. pelvic pain)
Almeida et al. 2016, Brazil [26]	To investigate occurrence of symptoms of PFD among young female nulliparous athletes and nonathletes, paired by BMI. Also, influence of sport modality on occurrence and severity of urinary dysfunctions	Cross-sectional study	163 (67 athletes & 96 non-athletes, paired by BMI)	Volleyball, swimming, judo, artistic gymnastics trampoline. Nulliparous	Age range = 15 to 29 years Mean age (± SD) years - athletes 18 (± 5), non-athletes 21 (± 4)	**Questionnaire:** background information, sports practice and UI questions Included questions *extracted from* ICIQ-UI-SF, ISI, FSFI, Rome III, ICIQ-VS	Descriptive statistics Mann-Whitney test, chi-squared test, Fischer exact test, odds ratio	**a. UI** - 52.2% Overall prevalence-52.2% (n = 35/67) **b. ARD** AI (flatus)-64.6% (n = 42/67), AI (faeces)- 0% Constipation- 68.2% (n = 45/67) **c. SD -** dyspareunia 13.8% (4/29), vaginal laxity - 13.8% (4/29) **d. POP** -0% reported
Cardoso et al. 2018, Brazil [27]	To evaluate the prevalence of UI in female athletes practising high-impact sports and its association with knowledge, attitude, and practice	Observational and knowledge, attitude and practice survey	n = 118	Athletics, handball, Volleyball, basketball, futsal and judo Nulliparous	Mean age (± SD) years = 21.6 (± 2.7). ≥ 18 years and ≤ 30 years	**Questionnaire:** background information, sports practice and UI questions ICIQ-UI-SF KAP survey	Descriptive statistics Chi-squared test, univariate & multivariate analysis, odds ratio	**a. UI -** 70.0% Overall Prevalence -70.0% (82/118) MUI - 54% (44/82) - most common SUI - 23% (19/82) UUI - 23% (19/82) UI during practice - 61% (50/82) UI during competition - 45% (37/82)
Carls 2007, USA [23]	To identify the prevalence of stress incontinence (SUI) in young female athletes and assess the need for preventative UI education	Cross-sectional study	N = 86 (34 school athletes & 52 college athletes)	Basketball, track, softball, volleyball, cheerleading, weightlifting, pompom dance Nulliparous.	Age range = 14 to 21 years Average age 17	**Questionnaire:** BFLUTS revised to include questions pertaining to sports and educational needs	Descriptive statistics calculated by hand	**a. UI** SUI - 28% (24/86) UUI - 7%
Carvalhais et al. 2018, Portugal [28]	To evaluate the prevalence of UI in female elite athletes of different sports compared to age-matched controls and to investigate possible risk factors for UI	Cross-sectional study	N = 744 (372 elite athletes & 372 age-matched controls)	Variety (n = 28) Sub-divided (G1 technical G2 endurance, G3 aesthetic G4 weight G5 Ball games, G6 Power G7 gravity) Nulliparous and parous	Age range = 15 to 48 years. Median age = 19 years for both athletes and controls	**Questionnaire**: Section 1 - background information; Section 2 - medical, obstetric & gynaecological and UI history Section 3 - sports practice and UI - ICIQ-UI-SF	Descriptive statistics Mann-Whitney test, chi-squared test, Fischer exact test, binary logistic regression, odds ratio	**a. UI** - 29.6% Overall prevalence (110/372) SUI - 19.6% most common (73/372) UUI - 3.8% (14/372) MUI - 5.9% (22/372) Other UI - 0.3% (n = 1/372) **b. ARD** constipation - median 33. IQR 8.9
Carvalho et al. 2020, Brazil [29]	To investigate the occurrence of urinary, anal, sexual and POP symptoms as well as symptoms originating from premenstrual syndrome in women who practice cheerleading	Cross-sectional study	N = 156 (78 cheerleaders & 76 nonathletes)	Athletes - high-performance cheerleaders, nulligravida, normal weight (BMI between 18.5 and 24.9 kg/m^2^) Control group - nonathletic female university students Nulliparous	Age range = 15 to 29 years, mean age (± SD) years - athletes 20.8 (± 2.3), non-athletes 21.9 (± 2.4)	**Questionnaire**: background information, sports practice and UI questions ICIQ-UI-SF Questions *extracted from* KHQ, FISI, FSFI, ICIQ-VS, MSQ	Descriptive statistics Shapiro-Wilk test (to confirm normality of data), Mann-Whitney test, odds ratio	**a. UI** - 26.9% UI overall prevalence 26.9% (21/78) UI - 47.6% (10/21) during training SUI - 66.7% (14/21) was the most prevalent UUI - 9.5% (2/21) MUI - 23.8% (5/21) **b. ARD** AI - 62.8% (49/78) and was most prevalent symptom of PFD Flatus - 55.1% (43/78) Constipation - 25.6% (20/78) **c. SD** dyspareunia - 53.8% (40/71) Vaginismus - 2.8% (2/71) Dysmenorrhea - 92.3% (72/78) **d. POP**-7.7% (6/78)
Caylet et al. 2006, France [30]	To assess the prevalence of UI in elite athletes versus the general population and to analyse the condition of occurrence of urine loss	Survey- epidemiological athlete/ non-athlete study	N = 583 (157 elite athletes & 426 controls)	Athletes from volleyball, swimming, rugby, handball, basketball, football, 'other'. Controls from ‘Physicians & Occupational Networks’. Nulliparous and parous	Age range = 18 to 35 years Mean age (± SD) years - athletes 23.37 (± 4.5), non-athletes 25.06 (± 4.6)	**Questionnaire**: A validated survey for use at a hospital clinic. Medical, obstetrical, gynaecological history, physical activities, including type of sports and duration of activities and UI	Descriptive statistics Student’s t-test, Kruskal-Wallis test, chi-squared test, Fischer exact test, McNemar test	**a. UI** - 28% UI Overall prevalence 28% (44/157) SUI - 41.9% (18/44) - was the most prevalent UUI - 34.9% (15/44) MUI - 23.2% (10/44)
Da Roza et al. 2015, Portugal [31]	To investigate in young nulliparous female trampolinists hypothetical associations between the level of athletic performance and the volume of training with urine leakage	Cross-sectional cohort study	N = 22	Trampolinists, classified as elite and non-athlete. Nulliparous	Age range = 14 to 25 years, mean age (± SD) - 18.1 (± 2.4)	**Questionnaire:** background information, sports practice and UI questions ICIQ-UI-SF	Descriptive statistics Shapiro-Wilk test, Kruskal-Wallis H test, Spearman rank correlation test	**a. UI** - 72.7% (16/22) **b**. **ARD** Constipation - 0%
Dobrowolski et al. 2020, Canada [32]	To determine prevalence, impact and management of stress urinary incontinence (SUI) among rope-skipping (RS) athletes. First survey aimed at identifying prevalence, impact and management strategies of SUI among current RS athletes. Second to determine whether SUI contributes to athletes’ decisions to retire from RS participation	Cross-sectional observational study	Survey 1 (current RS athletes) N = 103 (89 females & 14 males), survey 2 (retired RS athletes) N = 77 (74 females & 3 males)	Rope-skipping (RS) athletes, current and retired Nulliparous and parous	Female age range = 13 to 59 years, median age (IRQ) - 16 (15–21) years, male age range = 13 to 35 years, median age (IRQ) - 18 (13–28) years	**Questionnaire:** Survey 1 - background information, 11-point Likert scale regarding UI interference with rope skipping, ICIQ-UI-SF and *an* *unvalidated sport-specific questionnaire inspired by the* IIQ-7 Survey 2 - Questionnaire asking reasons for retiring from RS	Descriptive statistics Odds ratio	**prevalence in female RS:** **a. UI** SUI - 75% (67/89) **b. ARD** constipation - 12% (10/87)
Dockter et al. 2007, USA [33]	To determine the prevalence of UI in female collegiate athletes as compared to the prevalence in age-matched controls and to determine if there was a difference in the prevalence of UI among various sporting activities. Also examined strategies to prevent or manage UI in this population	Prospective cross-sectional survey	N = 177 (109 female collegiate athletes & 68 non-athletic controls)	Athletes competing in track and field athletics, basketball, soccer, softball, volleyball & cheerleading Nulliparous and parous	Age range = 18 to 25 years, mean age (± SD) years - athletes 19.17 (± 1.04), non-athletes 18.82 (± 0.75)	**Questionnaire**: Questionnaire designed for the purpose of the study	Descriptive statistics Independent t-test, chi-squared test	**a. UI** SUI - UL during coughing, sneezing and/or laughing - 46.8% (51/109); UL during physical activity - 40.4% UUI - 29.6%
Eliasson et al. 2002, Sweden [35]	To survey the prevalence of stress urinary incontinence in female elite trampolinists	Cross-sectional study	N = 35	Trampolinists Nulliparous	Age range = 12 to 22 years, mean age 15 years	1. **Questionnaire:** Questionnaire designed for the purpose of the study **2. Pad test** **3**. **PFM strength** by perineometer	Descriptive statistics Mann-Whitney test, Spearman’s rank correlation test	**a. UI -** 80% SUI -overall prevalence 80% (28/35) reported SUI, but only during trampoline training
Eliasson et al. 2008, Sweden [34]	To describe the occurrence of urinary leakage in young and mostly nulliparous women with a history of regular organised trampoline training as adolescents and to identify possible risk factors	Cross-sectional study	N = 305 [85 'competition' group (CG) & 220 'recreational' group (RG)]	Female ex-trampolinists in Sweden with licence for trampolining between 1995–1999 Nulliparous and parous	Median age = 21 (range 18–44) years	**Questionnaire**: Questionnaire designed and validated for study of UI in young nulliparous/primiparous women	Descriptive statistics Mann-Whitney test, chi-squared test, logistic regression	**a. UI -** overall = 68% Total of 68% (209/305) of trampolinists reported UL SUI -45% (138/305) reported UL when trampolining Current UI - CG - 57% (48/85); RG - 48% (106/120) **b. ARD** constipation - overall = 14.1% (43/305) CG - 13% (11/85): RG - 15% (32/120)
Faulks & Catto 2021, Australia [36]	To establish the prevalence of SUI among elite female rugby union players within Australia. Study further analysed rates of SUI between participants playing in the forward and back positions, self-reported consequences of SUI on athletic performance during a game or training session and the percentage who believed SUI to be a reason for withdrawing from rugby union in the future	Cross-sectional study	N = 65	Elite female rugby union players from the Australian Rugby Union Nulliparous and parous	Mean age (± SD)- 26 (± 5.1) years	**Questionnaire**: Modified version of the QUID	Descriptive statistics Chi-squared test	**a. UI** SUI - 60% (39/65) during a game or training
Ferreira et al. 2014, Portugal [37]	To verify the effectiveness of the pelvic floor muscle rehabilitation programme (PFMRP) in female volleyball athletes, analysing the amount and frequency of urinary leakage	Experimental	N = 32 [16 experimental group (EG) & 16 control group (CG)]	Volleyball athletes with symptoms of SUI Nulliparous	Mean age ± SD (range) years - EG 19.4 ± 3.24 (16–25); CG 19.1 ± 2.11 (17–26)	**1.Questionnaire**: Baseline information **2. Pad test** **3. Urinary diary** assessment of frequency of UL	Descriptive statistics Shapiro-Wilk, Student’s t-test, chi-squared test, Fischer exact test	**a. UI** All participants in the study reported symptoms of SUI
Gram & Bo 2020, Norway [38]	To investigate the prevalence and risk factors for UI in rhythmic gymnasts and the impact of UI on performance and their knowledge of the pelvic floor and PFM training	Cross-sectional study	N = 107	Rhythmic gymnasts Nulliparous	Age range = 12 to 21 years, mean age (± SD) years - 14.5 (± 1.6)	**1.Questionnaire**: Background information, influence of UI on sports practice, ICIQ-UI-SF Triad-specific self-report questionnaire LEAF-Q **2. Clinical hypermobility testing**	Descriptive statistics Kolmogorov-Smirnov, Shapiro-Wilk, Student’s t-test, chi-squared test, Fischer exact test, logistical regression	**a. UI -** 31.8% UI was reported in 31.8% (34/107) athletes of which: SUI - 61.8% was most reported (21/34) and of those reporting SUI, 57.1% reported leakage only during physical activity UUI - 8.8% (3/34) MUI - 17.6% (6/34) No obvious reason - 11.8% (4/34)
Hagovska et al. 2018, Slovakia [39]	To determine the prevalence of SUI symptoms in sportswomen (high-intensity physical activity) and non-sportswomen (low-intensity physical activity), according to estimated intensity of physical activity in metabolic equivalents using the IPAQ questionnaire. Another goal was to identify relationships among SUI symptoms, intensity of PA & QOL	Cross-sectional study	N = 557 (270 sportswomen & 287 non-sportswomen)	Women from university sport clubs at national levels. No further detail regarding types of sport Nulliparous	Mean age all (± SD) years 20.9 (± 2.8), sportswomen 20.7 (± 3.3); non-sportswomen 21.1 (± 2.3)	**Questionnaire**: Questionnaires used- ICIQ-UI-SF OAB-q I-QoL IPAQ	Descriptive statistics Logistic regression, odds ratio, Pearson correlation coefficient	**a. UI -** 6.14% ICIQ-UI SF confirmed slight UL in 6.14% (n = 33/270) sportswomen
Jácome et al. 2011, Portugal [40]	To investigate the prevalence of UI in a group of female athletes and to explore the impact on their lives by identifying their emotions regarding urine loss and techniques used to reduce UI episodes	Cross-sectional survey & focus group	N = 106	Track and field athletes, basketball, indoor football Nulliparous and parous	Mean age (± SD) = 23(± 4.4) years. Range not given. Inclusion criteria for age ≥ 18years and < 45years	**1.Questionnaire**: (N = 106) Background information/sports practice; UL characterisation; risk factors for UI **2. Focus group** (N = 7) Non-directive manner, semi-structured guide	Descriptive statistics Binomial test, chi-squared test, Fischer exact test, 2-way ANOVA, thematic analysis	**a. UI -**41.5% That 41.5% (44/106) of athletes had experienced UI at least once. *Of those experiencing UI* SUI - 61.4% (27/44) UUI - 20.5% (9/44) MUI - 18.2% (8/44)
Larsen & Yavorek 2006, USA [41]	To evaluate both baseline pelvic support and incontinence in relation to physical activity in nulliparous college age women	Prospective observational study	N = 144	Women at the US Military Academy College athletes Nulliparous	Mean age = 19.6 years	**1. Questionnaire**: Questionnaire designed for the purpose of the study **2. POPQ**	descriptive statistics	**a. UI -** 19.4% Urinary incontinence was reported by 28/144 (19.4%) of the female athletes. SUI -43%, (12/28) UUI – 28.5% (8/28) MUI – 28.5% (8/28) **d. POP**-50% with 46% (66/144) having stage I and 4% (6/144) having stage II pelvic support
Ludviksdottir et al. 2018, Iceland [42]	To measure and compare PFM strength in competition-level athletes and untrained women and evaluate the women's ability to contract the PFMs correctly and to explore frequency of SUI and assess the women's knowledge and awareness of PFMs	Case control study	N = 34 (18 athletes & 16 untrained women)	Various sports (handball, soccer, gymnastics, badminton, weightlifting, bootcamp, crossfit) Nulliparous	Women, age range = 18 to 30 years. Mean age (± SD) years = athletes 24.2 (± 3.2), untrained 24.1 (± 2.9) Nulliparous	**1. Questionnaire**: Background information, exercise training, knowledge of PFMs ICIQ-UI-SF **2. PFM strength measurement** - by Myomed 932 pressure sensor 2.	Descriptive statistics T-test, odds ratio, binary logistical regression	**a. UI -** 61.1% Urinary incontinence was reported by 11/18 (61.1%) of the female athletes (reported as occurring during high-intensity exercise and was therefore assumed to be SUI)
Nygaard et al. 1994, USA [44]	To determine the prevalence of the symptom of urinary incontinence during athletic endeavours among a group of nulliparous, elite college varsity female athletes	Cross-sectional study	N = 144	Gymnastics, volleyball, swimming, field hockey, softball, basketball, golf, track athletics, tennis Nulliparous	Mean age (± SD) = 19.9 (± 3.3) years	**Questionnaire**: Questionnaire designed for the purpose of the study	Descriptive statistics Student’s t-test, chi-squared test, Fisher exact test	**a. UI - **49% UI was reported by 49% (71/144) of the female athletes 28% (40/144) athletes admitted to at least one episode of UI while practicing or competing in their sport. Other bladder symptoms noted during competition included: Urgency - 31% Increased frequency - 37% Bladder pain - 7%
Nygaard 1997, USA [43]	To determine whether women engaged in strenuous, provocative exercise are more likely to be incontinent in future life than similarly fit women who participated in less provocative exercise	Retrospective cohort study	N = 104	Ex-Olympian low-impact (swimming) and high-impact sports (gymnastics, track & field athletics). Nulliparous and parous	Mean age (range) years - low-impact group 42.4 (30–54), high-impact group 46.2 (30–63)	**Questionnaire**: Questionnaire designed for the purpose of the study	Descriptive statistics Two-tailed t-test, chi-squared test, Fisher's exact, Wilcoxon rank sign, logistic regression	**a. UI - during Olympic sporting activity** Total of 35.8% (19/53) high-impact athletes (3 could not recall) and 4.5% (2/44) low-impact athletes, 4 could not recall (swimmers) reported having UI during their time as Olympians **Currently - **SUI - 41.1% (23/56) high-impact athletes and 50% (24/48) low-impact athletes (swimmers) reported SUI currently UUI - 33.9% (19/56) high-impact athletes and 16.7% (8/48) low-impact athletes (swimmers) reported UUI currently
Pires et al. 2020, Portugal [53]	To investigate the effects of pelvic floor muscles training in elite female volleyball athletes and whether it is an effective therapy for stress urinary incontinence	Randomised controlled trial	N = 13 athletes (7 in experimental group (EG) & 6 in control group (CG)	Volleyball athletes Nulliparous	Mean age ± SD (range) years - experimental group 22.7 1± 4.99 (18–30), control group 21.83 ± 5.19 (18–31)	**1.Questionnaire**: Questionnaire background sociodemographic and anthropometric data, KHQ **2. Pad test** **3**. **Pelvic floor muscle strength measurement** by perineometer	Descriptive statistics Shapiro-Wilk test, Levene, M Box test, Student’s t-test, ANOVA	**a. UI (SUI) -** 61.5% UI was reported by 61.5% (8/13) of the female athletes at initial evaluation (both CG & EG)
Poswiata et al. 2014, Poland [24]	To determine the prevalence of SUI in a group of elite female endurance athlete. Also, to compare the SUI rates in the groups of female cross-country skiers and runners to determine whether the training weather conditions like temperature and humidity might also influence the prevalence of UI	Cross-sectional study	N = 112 Female endurance athletes (57 cross-country skiers and 55 runners)	Cross-country skiers Runners Nulliparous	Mean age (± SD) years = cross-country skiers 26.61 (± 4.41), runners 29.49 (± 6.02).	**Questionnaire**: Short form of the UDI-6	Descriptive statistics Chi-squared test	**a. UI -** 50% UI was reported by 56/112 (50%) of the female athletes SUI -45.54% (51/112) UUI - 27.68% (31/112) MUI - 18.75% (21/112) Frequency - 58.04% (65/112) Bladder-emptying problems - 33.04% (37/112) **e. Pelvic pain** pain or discomfort in lower abdominal or genital area 36.61% (41/112) **Overall -** no statistically significant differences were noted between the groups
Rodríguez-López 2021, Spain [8]	To determine the prevalence of urinary incontinence (UI) among elite athletes and to compare prevalence between sexes and across different sports modalities	Observational, cross-sectional study	N = 455 female athletes (total N = 754 elite athletes; females n = 455 and males n = 299)	Wide variety (38 sports) Examples: athletics, soccer, gymnastics, rugby, judo, swimming, hockey, karate, orienteering dance, other sports Nulliparous and parous	Mean age (± SD) years = female athletes 23.18 ±7.10 (Overall - 23.04 ± 7.16, males - 22.81 ± 7.26)	**Questionnaire**: Background information - anthropometric data, medical history, sports practice, and UI data ICIQ-UI-SF 3IQ ISI	Descriptive statistics Shapiro-Wilks Student’s t-test chi-squared test Odds ratio (OR) Pearson’s coefficient	**a. UI -** 45.1% UI was reported by 205/455 (45.1%) of the female athletes SUI - 66% (135/205) UUI - 16% (33/205) MUI - 4% (8/205) **b. ARD** constipation - 13.2% (n = 60/455)
Sandwith & Robert 2021, Canada [45]	To determine the prevalence of urinary incontinence among female university varsity rugby players. Secondary objectives are to understand when the incontinence occurred and to assess the degree of bother experienced	Cross-sectional study	N = 95	Female rugby players Nulliparous	Mean age (± SD) years = 19.9 ± 1.8	**Questionnaire**: Background information - age, height, weight and hours spent training was obtained A specific rugby-related activity questionnaire was developed. Degree of bother, previous treatment, desire for treatment, when incontinence occurred and coping strategy UDI -6	Descriptive statistics. Unpaired t-test Linear regression	a. UI - 54% UI was reported by 51/95 (54%) of the female athletes SUI - 82% (42/51) UUI - 39% (20/51) Bladder-emptying problems - 16% (8/51) **e. Pelvic pain** pain or discomfort in lower abdominal or genital area 26% (13/51)
Skaug et al. 2020, Norway [46]	To investigate prevalence and risk factors for PFD powerlifters and Olympic weightlifters. Furthermore, to investigate the impact and bother of PFD and knowledge of the PFM	Cross-sectional study	N = 180 females (total N = 384 females n =180 and males n = 204)	Top national & international level male & female powerlifters & Olympic weightlifters Nulliparous and parous	Mean age weightlifters SD (range) years - female lifters 31.0 weightlifters 10.7 (18–65) (Males - 34.0 weightlifters 13.5 (18–84))	**Questionnaire**: Background information - age, body mass index [BMI], parity, training frequency, level of competition, years specialising in sport, generalised hypermobility, straining at toilet, female athlete triad, knowledge of PFM ICIQ-UI-SF Questions from: ICIQ-B for AI, ICIQ-V for POP LEAF-Q	Descriptive statistics Shapiro-Wilks Odds ratio (OR) Logistic regression	a. UI female athletes - 50% UI was reported by 90/180 (50%) of the female lifters SUI - 41.7% (75/180) UUI - 1.7% (3/180) MUI - 3.9% (7/180) **b. ARD female athletes -** 80% AI - 80% (144/180) Liquid - 32.8% (59/180); solid - 7.2% (13/180); gas - 76.7% (138/180) **Straining to defecate - female athletes** Never - 9.4% (17/180); rarely - 39.4% (71/180); some of the time - 46.7% (84/180); most of the time - 3.9% (7/180); always - 0.6% (1/180) **d. POP**-23.3% (42/180)
Skaug et al. 2022, Norway [47]	To investigate the prevalence of and risk factors for UI and AI in high-performance female artistic gymnasts, team gymnasts and cheerleaders and to investigate the bother of UI and AI, influence of SUI on sport performance and the athletes’ knowledge of the pelvic floor muscles (PFM)	Cross-sectional study	N = 319 (n = 68 artistic gymnasts, n = 116 team gymnasts, n = 135 cheerleaders)	Artistic gymnasts, team gymnasts, cheerleaders at the top national junior and senior level in Norway Parity not assessed because of young age	Mean age ± SD (range) years - All athletes - 17.4 ± 3.2 (12–36) Artistic gymnasts 16.8 weightlifters 3.6 (12–36) Team gymnasts 17.1 ± 2.7 (13–28) Cheerleaders 17.9 ± 3.3 (12–29)	**Questionnaire**: Background data, medical and sport practice, knowledge of PFM ICIQ-UI-SF Questions from: ICIQ-B for AI, LEAF-Q	Descriptive statistics Logistic regression Odds ratio (OR) Chi-squared test	**a. UI - Overall UI-** 67.4% UI was reported by 215/319 (67.4%) of the athletes SUI - 63% (201/319); UUI - 11.6% (31/319); MUI - 9.4% (7/215) **UI artistic gymnasts - 70.6**% Urinary incontinence was reported by 48/68 (70.6%) of the artistic gymnasts SUI- 70.6% (48/68); UUI - 8.8% (6/68); MUI - 8.8% (6/68) **UI team gymnasts - 83.6**% UI was reported by 97/116 (83.6) of the team gymnasts SUI- 80.2% (93/116); UUI - 12.9% (15/116); MUI -11.2% (13/116) **UI cheerleaders - 51.9**% UI was reported by 70/135 (51.9%) of the cheerleaders SUI - 44.4% (60/135); UUI - 11.9% (16/135); MUI - 8.1% (11/135) **Straining to void - all athletes** Never - 36.1% (115/319); occasionally - 45.5% (145/319); frequently - 14.1% (45/319); daily - 4.4% (14/319) **b. ARD** **overall AI - 84**% AI was reported by 268/319 (84%) of the athletes Liquid - 40.8% (130/319); solid - 12.2% (39/319); gas - 81.2% (259/319) **AI artistic gymnasts - 82.4**% (56/68) Liquid - 36.8% (25/68); solid - 11.8% (8/68); gas - 82.4% (56/68) , **AI - team gymnasts - 86.2**% (110/116) Liquid - 41.4% (48/116); solid - 13.8 % (16/116); gas - 83.6% (97/116) **AI - cheerleaders 83**% (112/135) Liquid - 42.2% (57/136); solid - 11.1% (15/135); gas - 78.5% (106/135); **straining to defecate - all athletes** Never - 3.1% (10/319); rarely - 34.5% (110/319); some of the time - 52.4% (167/319); most of the time- 9.7% (31/319); always - 0.3% (1/319)
Thyssen et al. 2002 Denmark, [25]	To determine the frequency of urinary loss in elite women athletes and dancers	Cross-sectional study	N = 291	Badminton Handball Basketball Volleyball Athletics Gymnastics Aerobics Ballet Nulliparous and parous	Average age (range) years – 22.8 (14–51)	**Questionnaire**: Questionnaire designed for the purpose of the study	Descriptive statistics McNemars test	**a. UI** - 51.9% A total of 151/291 (51.9%) had experienced urine loss while participating in their sport or in daily life situations. **UI during sport -** 43% (125/291) experienced urine loss while participating in their sport
Velázquez-Saornil et al., Spain 2021 [48]	To assess the prevalence of UI in federated female athletes (federative sports license). In addition to observing urine leakage in the study subjects according to the sport discipline of athletics and age, the methods of containment, the characteristics of leakage and the risk factors that may lead to UI were analysed. Biopsychosocial component of UI in this group of athletes was also analysed	Cross-sectional study	N = 63	Athletics 5 categories: 1.Long-distance running 2.Speed events 3. Middle distance races 4. Jumping events 5. Throwing events Nulliparous and parous	Mean age ± SD (range) years – 30.78 ± 12.16 (18–61)	**Questionnaire**: KHQ ICQ-UI SF	Descriptive statistics Chi-squared test Student’s t-test	**a. UI** - 44.4% 28/63 women (44.4%) reported UI SUI -89.3 %, (25/28) (25% reported coughing and sneezing and 64.3% reported coughing and sneezing during physical exercise)
Wikander et al. 2019, Australia [49]	To determine the prevalence of UI in competitive women powerlifters and establish if commonly cited risk factors affect the incidence of UI	Cross-sectional study	N = 134	Competitive powerlifters Information regarding parity not collected	Age range 20–59 years	**Questionnaire**: Background information with sports-specific questions ISI	Descriptive statistics Kruskal-Wallis ANOVA, Cohen's *f,* Kendall's tau B Thematic analysis of comments section	**a. UI** - 41% UI had been experienced by 41% (55/134) of women powerlifters at some stage in life, 37% (50/134) during lifting activities 11% (15/134) during everyday activities
Wikander et al. 2020, Australia, UK, USA, Canada & New Zealand [50]	To determine the prevalence of urinary and athletic incontinence and establish which activities and contexts were most likely to provoke urine leakage in women CrossFit competitors	Cross-sectional study	N = 452	Women CrossFit competitors Nulliparous and parous	Mean age ± SD (range) years – 36 ± 9 (20–63)	**Questionnaire**: Developed specifically for CrossFit participants. Focus on context in which UI occurred and exercises most likely to cause UI ISI	Descriptive statistics	**a. UI** - 46% 46% (N = 208/452) of women experienced UI at some point in their life and 41.8% (N = 189/452) reported having experienced UI in the last 3 months prior to the study
Wikander et al. 2021A Australia, UK, USA, Canada & New Zealand [51]	To determine the prevalence of UI in competitive women powerlifters; identify possible risk factors and activities likely to provoke UI and establish self-care practices	Cross-sectional study	N = 480	Women powerlifters Nulliparous and parous	Mean age ± SD (range) years – 35 ± 10 (20–89)	**Questionnaire**: Background information Questions to identify actions and events associated with UI, self-care strategies ISI	Descriptive statistics Kendall’s tau-b Eta correlation coefficient	**a. UI** - 48.8% 48.8% (234/480) of participants in this study reported experiencing UI at some point in their lifetime 43.9% (211/480) had experienced UI in the 3 months prior to the study
Wikander et al. 2021B Australia & other English-speaking countries (not specified) [52]	To explore multifactorial issue of UI in competitive women weightlifters focus on prevalence, risk factors and activities provoking UI. In addition to identify self-care strategies used by incontinent competitive women weightlifters. Finally, confidence in performing PFE & utilization of women’s health professionals	Cross-sectional study	N = 191	Women weightlifters Nulliparous and parous	Mean age ± SD years- 35.92 ± 12 (20–89)	**Questionnaire**: Questionnaire Background information Question to identify actions and events associated with UI, self-care strategies ISI	Descriptive statistics Pearson’s correlations	**a. UI** - 36.6% 36.6% (70/191) of women had experienced UI at some point in their life, 31.9% (61/191) reported having experienced UI during the previous 3 months

*****BFLUTS = Bristol Female Lower Urinary Tract Symptoms Questionnaire; FISI = Fecal Incontinence Severity Index; FSFI = Female Sexual Function Index; ICIQ-UI-SF = International Consultation on Incontinence Questionnaire-Short Form; ICIQ-VS = International Consultation on Incontinence Questionnaire-Vaginal Symptoms; IIQ-7 = Incontinence Impact Questionnaire; IPAQ = International Physical Activity Questionnaire; I-QoL = International Quality of Life Questionnaire; ISI = Incontinence Severity Index; KAP = Knowledge Attitude and Practice; LEAF-Q = Low Energy Availability in Females Questionnaire; KHQ = King’s Health Questionnaire; MSQ = Menstrual Symptom Questionnaire; OAB-q = Overactive Bladder Questionnaire; POPQ = Pelvic Organ Prolapse Quantification; QUID = Questionnaire for Urinary Incontinence Diagnosis; Rome III = Rome III Questionnaire; 3IQ = Three Incontinence Questions Questionnaire

**PFD = Pelvic Floor Dysfunction; UI = urinary incontinence; SUI = stress urinary incontinence; UUI = urge urinary incontinence; MUI = mixed urinary incontinence; UL = urinary leakage; ARD = anorectal dysfunction; AI = anal incontinence; POP = pelvic organ prolapse; PFM = pelvic floor muscle; SD = sexual dysfunction)

## References

[1] Haylen BT, de Ridder D, Freeman RM, Swift SE, Berghmans B, Lee J (2010). An International Urogynecological Association (IUGA)/International Continence Society (ICS) joint report on the terminology for female pelvic floor dysfunction. Int Urogynecol J.

[2] Bo K, Frawley HC, Haylen BT, Abramov Y, Almeida FG, Berghmans B (2017). An International Urogynecological Association (IUGA)/International Continence Society (ICS) joint report on the terminology for the conservative and nonpharmacological management of female pelvic floor dysfunction. Neurourol Urodyn.

[3] Amaral MO, Coutinho EC, Nelas PA, Chaves CM, Duarte JC (2015). Risk factors associated with urinary incontinence in Portugal and the quality of life of affected women. Int J Gynaecol Obstet.

[4] Pizzol D, Demurtas J, Celotto S, Maggi S, Smith L, Angiolelli G (2021). Urinary incontinence and quality of life: a systematic review and meta-analysis. Aging Clin Exp Res.

[5] Mallah F, Montazeri A, Ghanbari Z, Tavoli A, Haghollahi F, Aziminekoo E (2014). Effect of Urinary Incontinence on Quality of Life among Iranian Women. J Family Reprod Health.

[6] Mendes A, Hoga L, Gonçalves B, Silva P, Pereira P (2017). Adult women's experiences of urinary incontinence: a systematic review of qualitative evidence. JBI Database System Rev Implement Rep.

[7] Nygaard IE, Shaw JM (2016). Physical activity and the pelvic floor. Am J Obstet Gynecol.

[8] Rodríguez-López ES, Calvo-Moreno SO, Basas-García Á, Gutierrez-Ortega F, Guodemar-Pérez J, Acevedo-Gómez MB (2021). Prevalence of urinary incontinence among elite athletes of both sexes. J Sci Med Sport.

[9] Almousa S, Bandin Van Loon A (2019). The prevalence of urinary incontinence in nulliparous female sportswomen: A systematic review. J Sports Sci.

[10] de Mattos Lourenco TR, Matsuoka PK, Baracat EC, Haddad JM. Urinary incontinence in female athletes: a systematic review. Int Urogynecol J. 2018;29(12):1757-63. 10.1007/s00192-018-3629-z. 10.1007/s00192-018-3629-z 29552736

[11] Lourenco T, Matsuoka P, Baracat E, Doumouchtsis S, Haddad JM. Female athletes and urinary incontinence according to different intensities of impact: an updated systematic review with meta-analysis. ICS 2021 Melbourne Online; 2021; October 17^th^. Melbourne. https://www.ics.org/2021/abstract/98.

[12] Pires T, Pires P, Moreira H, Viana R (2020). Prevalence of Urinary Incontinence in High-Impact Sport Athletes: A Systematic Review and Meta-Analysis. J Hum Kinet.

[13] Teixeira RV, Colla C, Sbruzzi G, Mallmann A, Paiva LL (2018). Prevalence of urinary incontinence in female athletes: a systematic review with meta-analysis. Int Urogynecol J.

[14] Rebullido TR, Gómez-Tomás C, Faigenbaum AD, Chulvi-Medrano I (2021). The Prevalence of Urinary Incontinence among Adolescent Female Athletes: A Systematic Review. J Functional Morphol Kinesiol.

[15] Bø K, Sundgot-Borgen J (2010). Are former female elite athletes more likely to experience urinary incontinence later in life than non-athletes?. Scand J Med Sci Sports.

[16] Bø K, Nygaard IE (2020). Is Physical Activity Good or Bad for the Female Pelvic Floor? A Narrative Review. Sports Med.

[17] Moher D, Liberati A, Tetzlaff J, Altman DG (2009). Preferred Reporting Items for Systematic Reviews and Meta-Analyses: The PRISMA Statement. PLoS Med.

[18] Page MJ, McKenzie JE, Bossuyt PM, Boutron I, Hoffmann TC, Mulrow CD (2021). The PRISMA 2020 statement: an updated guideline for reporting systematic reviews. BMJ.

[19] Culleton-Quinn E, Fleming N, Mockler D, Cusack C, Bø K, Daly D. Female athletes’/sportswomen’s experiences of incontinence and pelvic floor symptoms: A systematic review. PROSPERO. 2020. Available from: https://www.crd.york.ac.uk/prospero/display_record.php?ID=CRD42020197330.

[20] Thomas J, Sutcliffe K, Harden A, Oakley A, Oliver S, Rees R, Brunton G, Kavanagh J (2003). Children and Healthy Eating: A systematic review of barriers and facilitators.

[21] Panda S, Begley C, Daly D (2018). Clinicians' views of factors influencing decision-making for caesarean section: A systematic review and metasynthesis of qualitative, quantitative and mixed methods studies. PLoS One.

[22] Braun V, Clarke V (2006). Using thematic analysis in psychology. Qual Res Psychol.

[23] Carls C (2007). The prevalence of stress urinary incontinence in high school and college-age female athletes in the midwest: implications for education and prevention. Urologic Nurs: Off J Am Urol Assoc Allied.

[24] Poswiata A, Socha T, Opara J (2014). Prevalence of stress urinary incontinence in elite female endurance athletes. J Human Kinet.

[25] Thyssen HH, Clevin L, Olesen S, Lose G (2002). Urinary incontinence in elite female athletes and dancers. Int Urogynecol J.

[26] Almeida MB, Barra AA, Saltiel F, Silva-Filho AL, Fonseca AM, Figueiredo EM (2016). Urinary incontinence and other pelvic floor dysfunctions in female athletes in Brazil: A cross-sectional study. Scand J Med Sci Sports.

[27] Cardoso AMB, Lima CROP, Ferreira CWS (2018). Prevalence of urinary incontinence in high-impact sports athletes and their association with knowledge, attitude and practice about this dysfunction. Eur J Sport Sci.

[28] Carvalhais A, Natal Jorge R, Bø K (2018). Performing high-level sport is strongly associated with urinary incontinence in elite athletes: a comparative study of 372 elite female athletes and 372 controls. Br J Sports Med.

[29] Carvalho C, da Silva Serrão PRM, Beleza ACS, Driusso P (2020). Pelvic floor dysfunctions in female cheerleaders: a cross-sectional study. Int Urogynecol J.

[30] Caylet N, Fabbro-Peray P, Marès P, Dauzat M, Prat-Pradal D, Corcos J (2006). Prevalence and occurrence of stress urinary incontinence in elite women athletes. Can J Urol.

[31] Da Roza T, Brandão S, Mascarenhas T, Jorge RN, Duarte JA (2015). Volume of training and the ranking level are associated with the leakage of urine in young female trampolinists. Clin J Sport Med.

[32] Dobrowolski SL, Pudwell J, Harvey MA (2020). Urinary incontinence among competitive rope-skipping athletes: a cross-sectional study. Int Urogynecol J.

[33] Dockter M, Kolstad AM, Martin KA, Schiwal LJ (2007). Prevalence of urinary incontinence: a comparative study of collegiate female athletes and non-athletic controls. J Women's Health Phys Ther.

[34] Eliasson K, Edner A, Mattsson E (2008). Urinary incontinence in very young and mostly nulliparous women with a history of regular organised high-impact trampoline training: Occurrence and risk factors. Int Urogynecol J.

[35] Eliasson K, Larsson T, Mattsson E (2002). Prevalence of stress incontinence in nulliparous elite trampolinists. Scand J Med Sci Sports.

[36] Faulks K, Catto T (2021). The prevalence of stress urinary incontinence among elite female rugby union players in Australia. Australian & New Zealand Continence J.

[37] Ferreira S, Ferreira M, Carvalhais A, Santos PC, Rocha P, Brochado G (2014). Reeducation of pelvic floor muscles in volleyball athletes. Rev Assoc Med Bras.

[38] Gram MCD, Bo K (2020). High level rhythmic gymnasts and urinary incontinence: Prevalence, risk factors, and influence on performance. Scand J Med Sci Sports.

[39] Hagovska M, Svihra J, Bukova A, Horbacz A, Svihrova V (2018). The impact of physical activity measured by the International Physical Activity questionnaire on the prevalence of stress urinary incontinence in young women. Eur J Obstet Gynecol Reprod Biol.

[40] Jácome C, Oliveira D, Marques A, Sá-Couto P (2011). Prevalence and impact of urinary incontinence among female athletes. Int J Gynecol Obstet.

[41] Larsen WI, Yavorek TA (2006). Pelvic organ prolapse and urinary incontinence in nulliparous women at the United States Military Academy. Int Urogynecol J.

[42] Ludviksdottir I, Hardardottir H, Sigurdardottir T, Ulfarsson GF (2018). Comparison of pelvic floor muscle strength in competition-level athletes and untrained women. Laeknabladid.

[43] Nygaard IE (1997). Does prolonged high-impact activity contribute to later urinary incontinence? A retrospective cohort study of female olympians. Obstet Gynecol.

[44] Nygaard IE, Thompson FL, Svengalis SL, Albright JP (1994). Urinary-incontinence in elite nulliparous athletes. Obstet Gynecol.

[45] Sandwith E, Robert M (2021). Rug-pee study: the prevalence of urinary incontinence among female university rugby players. Int Urogynecol J.

[46] Skaug KL, Engh ME, Frawley H, Bø K. Prevalence of Pelvic Floor Dysfunction, Bother and Risk Factors and Knowledge of the Pelvic Floor Muscles in Norwegian Male and Female Powerlifters and Olympic Weightlifters. J Strength Cond Res. 2020. 10.1519/JSC.0000000000003919. 10.1519/JSC.0000000000003919 33278274

[47] Skaug KL, Engh ME, Frawley H, Bø K (2022). Urinary and anal incontinence among female gymnasts and cheerleaders—bother and associated factors. A Cross-Sectional Stud Int Urogynecol J.

[48] Velázquez-Saornil J, Méndez-Sánchez E, Gómez-Sánchez S, Sánchez-Milá Z, Cortés-Llorente E, Martín-Jiménez A, et al. Observational study on the prevalence of urinary incontinence in female athletes. Int J Environ Res Public Health. 2021;18(11). 10.3390/ijerph18115591. 10.3390/ijerph18115591 PMC8197179 34073782

[49] Wikander L, Cross D, Gahreman DE (2019). Prevalence of urinary incontinence in women powerlifters: a pilot study. Int Urogynecol J.

[50] Wikander L, Kirshbaum MN, Gahreman DE (2020). Urinary incontinence and women crossfit competitors. Int J Women's Health.

[51] Wikander L, Kirshbaum MN, Waheed N, Gahreman DE. Urinary Incontinence in Competitive Women Powerlifters: A Cross-Sectional Survey. Sports Med - Open. 2021;7. 10.1186/s40798-021-00387-7. 10.1186/s40798-021-00387-7 PMC8651931 34874496

[52] Wikander L, Kirshbaum MN, Waheed N, Gahreman DE. Urinary Incontinence in Competitive Women Weightlifters. J Strength Cond Res. 2021. 10.1519/JSC.0000000000004052. 10.1519/JSC.0000000000004052 PMC9592169 34100787

[53] Pires TF, Pires PM, Moreira MH, Gabriel RECD, João PV, Viana SA (2020). Pelvic Floor Muscle Training in Female Athletes: A Randomized Controlled Pilot Study. Int J Sports Med.

[54] Nygaard I, Barber MD, Burgio KL, Kenton K, Meikle S, Schaffer J (2008). Prevalence of symptomatic pelvic floor disorders in US women. Jama.

[55] Swann C, Moran A, Piggott D (2015). Defining elite athletes: Issues in the study of expert performance in sport psychology. Psychol Sport Exerc.

[56] Williams A, Day S, Stebbings G, Erskine R (2017). What does 'elite' mean in sport and why does it matter?. The Sport and Exercise Scientist.

[57] Thomaz RP, Colla C, Darski C, Paiva LL (2018). Influence of pelvic floor muscle fatigue on stress urinary incontinence: a systematic review. Int Urogynecol J.

[58] Ree ML, Nygaard I, Bø K (2007). Muscular fatigue in the pelvic floor muscles after strenuous physical activity. Acta Obstet Gynecol Scand.

[59] Middlekauff ML, Egger MJ, Nygaard IE, Shaw JM (2016). The impact of acute and chronic strenuous exercise on pelvic floor muscle strength and support in nulliparous healthy women. Am J Obstet Gynecol.

[60] Pereira-Baldon VS, Avila MA, Dalarmi CB, de Oliveira AB, Driusso P (2019). Effects of different regimens for pelvic floor muscle training in young continent women: Randomized controlled clinical trial. J Electromyography Kinesiol: Off J Int Soc Electrophysiol Kinesiol.

[61] Dumoulin C, Cacciari LP, Hay-Smith EJC. Pelvic floor muscle training versus no treatment, or inactive control treatments, for urinary incontinence in women. Cochrane Database Syst Rev. 2018;10. 10.1002/14651858. 10.1002/14651858.CD005654.pub4 PMC6516955 30288727

